# Supplementary material for: Complexome profiling of the Chlamydomonas psb28 mutant reveals TEF5 as an early PSII assembly factor
Source: Plant Cell. 2025 Mar 17;37(6):koaf055. doi: 10.1093/plcell/koaf055 (PMC12164590; doi:10.1093/plcell/koaf055)
Supplement: koaf055_Supplementary_Data [file koaf055_supplementary_data.zip › psb28_interactive.zip!/plots/intensity/qualityPlots/peptides/OASTL4_Cre16.g685550.t1.2.html]

### Cre16.g685550.t1.2

Trivial name: OASTL4  
SeqCoverage [median]: 27.30  
Mass: 37.93 kDa  
  
 

| Sequence | MissedCleavage | Mass [Da] | wtIntensity | muIntensity | Score |
| --- | --- | --- | --- | --- | --- |
| AEEILASTPDAFMLQQFQNPNNPK | 0 | 2702.30 | 0.00 | 1043.92 | 55.13 |
| KSDVQLVAVEPAESPVLSGGKPGPHK | 2 | 2625.41 | 0.00 | 817.37 | 53.48 |
| LVVVVLPSFGER | 0 | 1313.77 | 2409.43 | 0.00 | 104.17 |
| VASRPENEGK | 1 | 1085.55 | 0.00 | 3626.80 | 82.17 |
|  |  |  | 2409.4 | 5488.1 |  |
